# The circadian rest-activity pattern predicts cognitive decline among mild-moderate Alzheimer’s disease patients

**DOI:** 10.1186/s13195-021-00903-7

**Published:** 2021-09-25

**Authors:** Adriano D. S. Targa, Iván D. Benítez, Faridé Dakterzada, John Fontenele-Araujo, Olga Minguez, Henrik Zetterberg, Kaj Blennow, Ferran Barbé, Gerard Piñol-Ripoll

**Affiliations:** 1 Translational Research in Respiratory Medicine, Hospital Universitari Arnau de Vilanova-Santa Maria, IRBLleida, Lleida, Spain; 2 Centro de Investigación Biomédica en Red de Enfermedades Respiratorias (CIBERES), Madrid, Spain; 3 Unitat Trastorns Cognitius, Clinical Neuroscience Research, Hospital Universitari Santa Maria, IRBLleida, Lleida, Spain; 4 Department of Physiology and Behavior, Federal University of Rio Grande do Norte, Natal, Brazil; 5 Department of Molecular Neuroscience, UCL Institute of Neurology, Queen Square, London, UK; 6 UK Dementia Research Institute, London, UK; 7 Institute of Neuroscience and Physiology, Department of Psychiatry and Neurochemistry, University of Gothenburg, Sahlgrenska University Hospital, Mölndal, Sweden; 8 Clinical Neurochemistry Laboratory, Sahlgrenska University Hospital, Mölndal, Sweden

**Keywords:** Alzheimer’s disease, Circadian rest-activity pattern, Intradaily variability, Neurofilament light, Cognitive decline

## Abstract

**Background:**

Alterations in circadian rhythms are present in the presymptomatic stage of Alzheimer’s disease (AD), possibly contributing to its pathogenesis. However, it is unknown whether such alterations are associated with worse outcomes once individuals are diagnosed with symptomatic disease. We aimed to evaluate the association between the circadian rest-activity pattern and AD-related features in patients with mild-moderate AD.

**Methods:**

We assessed the circadian rest-activity pattern of consecutive patients with mild-moderate AD through actigraphy for 14 days. Cerebrospinal fluid was obtained to determine the levels of important pathological markers including amyloid-beta protein (Aβ42), phosphorylated tau (P-tau), total tau (T-tau), and neurofilament light (NF-L). Neuropsychological evaluation was conducted at the beginning of the study and after 12 months of follow-up. Linear regression models were performed considering the global population and Aβ42+ patients only.

**Results:**

The cohort included 100 patients with mild-moderate AD. The median age [p_25_;p_75_] was 76.0 [73.0;80.0] years and 63.0% were female. Older age (effect size [SE] of 0.324 [0.096]; *p* = 0.001) and male sex (0.780 [0.193]; *p* = 0.001) were associated with increased fragmentation and decreased synchronization of the rhythm, respectively. After adjusting for age, sex, and season of the year, increased levels of T-tau (effect size [95% CI] of 0.343 [0.139 to 0.547]; *p* = 0.001) and NF-L (0.444 [0.212 to 0.676]; *p* = 0.001) were associated with a higher amplitude of the rest-activity rhythm. Increased fragmentation of the rhythm at baseline was associated with greater cognitive decline after one year of follow-up independent of age, sex, T-tau/Aβ42 ratio, educational level, and season of the year (− 0.715 [− 1.272 to − 0.157]; *p* = 0.013). Similar findings were obtained considering only the Aβ42+ patients.

**Conclusions:**

Our results suggest a potential role of the circadian rest-activity pattern in predicting the cognitive decline of patients with mild-moderate AD. Further studies are warranted to confirm these findings and to elucidate whether there is causality among the observed associations.

**Supplementary Information:**

The online version contains supplementary material available at 10.1186/s13195-021-00903-7.

## Introduction

Alzheimer’s disease (AD) is the most prevalent neurodegenerative disorder in the world and currently affects nearly 50 million people [1, 2]. This number is expected to increase given the age-associated risk and the growing number of older adults [2–4]. The pathological hallmarks of AD are the deposition of amyloid-beta protein (Aβ42), the formation of tau protein neurofibrillary tangles, and neurodegeneration [5]. Symptomatically, there is a progressive loss of cognitive function, which is often concomitant with behavioral symptoms, including depression, anxiety, hallucinations, sleep disturbances, and alterations in circadian rhythms [6–8].

Circadian rhythm alterations are mainly represented by the loss of a well-defined 24-h rest-activity pattern due to increased activity during the night and a decrease in activity during the day [9, 10]. Accordingly, sleep fragmentation and the presence of irregular bouts of sleep during the day decrease the amplitude and increase the fragmentation of the rest-activity rhythm [11, 12]. In addition, shifts in the bedtime and wake-up time to a later time in the day (i.e., phase delay) may occur in contrast to the usual advance of the phase observed in older adults [13, 14]. Furthermore, other circadian alterations can be observed, including those related to the secretion patterns of hormones such as melatonin and cortisol as well as in the daily fluctuations of body core temperature [15–17].

Alterations in circadian rhythms may precede the onset of classical cognitive symptoms in AD patients. A previous study demonstrated that cognitively unimpaired individuals with preclinical amyloid plaque pathology presented increased fragmentation of rest-activity rhythm independent of age and sex [10]. This suggests that circadian alterations could be used as biomarkers for the preclinical stage and/or contribute to the pathogenesis of the disease. Accordingly, prospective studies appear to support the idea that these alterations increase the risk of cognitive deterioration, leading to mild cognitive impairment and dementia [18–21]. Li and colleagues demonstrated that lower amplitude and higher fragmentation of the rest-activity rhythm increased the risk of developing AD in healthy older adults [8]. Similarly, these dysfunctions in addition to decreased interdaily stability independently increased the risk of such outcomes in individuals previously diagnosed with mild cognitive impairment [8]. However, it is unknown whether circadian alterations could be associated with worse outcomes once individuals have symptomatic AD.

To investigate this, we first performed a comprehensive characterization of the circadian rest-activity pattern of mild-moderate AD patients. In the sequence, we investigated whether the rest-activity rhythm could be associated with biomarkers of AD, such as Aβ42, phosphorylated tau (P-tau), total tau (T-tau), and neurofilament light (NF-L). Finally, we evaluated whether the rest-activity rhythm could predict the cognitive decline of mild-moderate AD patients at 12 months of follow-up.

## Methods

### Study population

This is an ancillary study of a prospective trial designed to evaluate the influence of obstructive sleep apnea (OSA) on the cognitive decline of AD patients after one year of follow-up (NCT02814045). Patients were recruited at the Cognitive Disorders Unit of the Hospital Universitari Santa Maria (Spain) for 4 years (2015-2019). The inclusion criteria comprised acetylcholinesterase inhibitor-naïve individuals aged over 60 years who were diagnosed with AD according to the National Institute on Aging and Alzheimer's Association (NIA-AA) clinical criteria [22]. Accordingly, to include patients with mild-moderate cognitive impairment, we only considered those with a Mini-Mental State Examination (MMSE) score ≥ 20. Secondary analyses were performed only with the patients who were Aβ42+ (values < 600 pg/ml were considered pathological amyloid deposition) or Aβ42- and had an MMSE score ≥ 20.

The exclusion criteria comprised (1) the presence of visual and/or communication problems that could make compliance with the study procedures difficult; (2) the presence of a previously diagnosed sleep disturbance; (3) the presence of excessive somnolence for unknown reasons; (4) comorbidities such as cancer, severe renal or hepatic insufficiency, severe cardiac or respiratory failure; (5) excessive alcohol intake (> 280 g/week); (6) MRI evidence of hydrocephalus, stroke, a space-occupying lesion, or any clinically relevant central nervous system disease other than AD; (7) the presence of mental disorders according to DSM-V-TR™ criteria; (8) any use of medications under investigation; (9) the presence of untreated (or treated for less than 3 months prior to the screening visit) vitamin B12 or folate deficiency; and (10) the presence of untreated thyroid disease.

The study was approved by the care ethics committee of Hospital Universitari Arnau de Vilanova (CE-1218) and conducted according to the Declaration of Helsinki. The patient, the responsible caregiver, and the legal representative (when different from the responsible caregiver) signed an informed consent form.

### Study design

After arriving at the Cognitive Disorders Unit of the Hospital Universitari Santa Maria (Spain), eligible patients were subjected to clinical evaluation for anthropometric data and sociodemographic data collection. Blood was obtained to determine apolipoprotein E (ApoE) genotypes, and cerebrospinal fluid (CSF) was collected to determine the levels of Aß42, T-tau, P-tau, and NF-L. In the sequence, the patients received a sleep log to be completed over 14 days as well as instructions related to the use of the actigraph that occurred during the same period. The neuropsychological evaluation was performed at the beginning of the study and after 12 months of follow-up.

### Clinical variables

The following variables were collected: age, sex, years of education, alcohol consumption, smoking, vascular risk factors (hypertension, diabetes mellitus, dyslipidemia, stroke, and cardiopathy), personal psychiatric history, and family psychiatric and neurological history. The body mass index (BMI) was calculated as body weight (in kg)/height (in m^2^).

### Apolipoprotein E (ApoE) genotype

DNA was extracted from buffy coat cells using a Maxwell® RCS blood DNA kit (Promega, USA). Twenty microliters of DNA were used for ApoE genotyping by polymerase chain reaction (PCR). ApoE genotype was dichotomized as ApoE-ε4 homozygous or heterozygous carrier (ApoEε4+) or not (ApoEε4−).

### CSF biomarkers

The CSF samples were collected at baseline between 8:00–10:00 a.m. They were placed in polypropylene tubes, centrifuged at 2000×*g* for 10 min at 4 °C, immediately frozen, and stored within 4 h in a − 80 °C freezer. The measurement of Aß42, T-tau, and P-tau was performed using commercial kits (Innotest® β-Amyloid-42; Innotest® hTAU Ag; and Innotest® Phospho-TAU181P, Fujirebio-Europe, Gent, Belgium). NF-L was measured by a commercial ELISA kit (Quidel, San Diego, CA). All determinations were performed in duplicate and in one round of experiments using one batch of reagents by board-certified laboratory technicians who were blinded to the clinical data. The intra-assay coefficients of variation were lower than 10% for internal quality control samples (two per plate). Based on previous data obtained by the research group, Aβ42 levels < 600 pg/ml were considered pathological [23].

### Circadian rest-activity pattern assessment

Rest-activity data were collected using a wrist-mounted actigraph (Actiwatch 2, Philips Respironics) for 14 days. Activity counts of 30-s epochs with a medium threshold for sensitivity were obtained and visualized using a software (Actiware 6.0.9, Philips Respironics). The data were revised with the support of a sleep log which was completed during the 14 days of actigraphy.

The following variables were obtained: time in bed (in minutes), total sleep time (in minutes), sleep efficiency (in %, defined as the ratio between total sleep time and the time spent in bed), latency (in minutes, defined as the time spent awake until the first sleep episode, which was represented by the first epoch of 10-min continuous stretch of immobility), and WASO (in minutes, defined as the time spent awake after sleep onset). In addition, different variables associated with the rest-activity rhythm were calculated from the 30-s epochs activity counts using the R software (version 3.4.2) [24, 25]. The intradaily variability represented the fragmentation of the rest-activity rhythm within each 24-h period, indicating whether there were daytime naps and/or nocturnal activity episodes. The interdaily stability represented how similar one 24-h period was to the next, indicating how synchronized the internal rest-activity rhythm was with the different zeitgebers (24-h light-dark cycle, food intake, physical activity) along the 14 days of actigraphy. L5 (the mean activity of the five consecutive hours with the lowest activity) and M10 (the mean activity of the ten consecutive hours with the highest activity) were used to calculate the relative amplitude (M10−L5/M10+L5). The relative amplitude represented the robustness of the rest-activity rhythm, indicating whether there was a difference in the magnitude of activity between active and rest phases.

### Neuropsychological assessment

Patients underwent a neuropsychological evaluation through the MMSE at the beginning of the study and after 12 months of follow-up. The MMSE includes questions to evaluate different domains, such as attention, time and place orientation, and word recall. The scores of this test range from 0 to 30, and a higher score indicates better cognitive function [26, 27].

### Statistical analysis

Descriptive statistics were performed to describe sociodemographic, clinical, and AD-related data. The absolute and relative frequencies were used for qualitative data and the medians (25th percentile; 75th percentile [p_25_;p_75_]) were estimated for quantitative variables. The normality of the distribution was assessed by the Shapiro-Wilk test. Intradaily variability and biomarkers values were log-transformed to normalize their distribution. The Bulging Rule transformation was used to normalize the relative amplitude [28].

The associations between rest-activity rhythm variables and sociodemographic, clinical, and sleep data were evaluated using linear models adjusted by age and sex. These analyses were performed including the global population. The associations between rest-activity rhythm variables and AD-related biomarkers were evaluated using linear models adjusted by age, sex, and season of the year [29]. These analyses were performed with the global population and stratified by amyloid groups (Aβ42+ and Aβ42− patients). Additional analyses were performed to evaluate the possible effect of sex on this context [30, 31]. The linear models were adjusted by age and season of the year and included the global population stratified by sex.

To investigate the association between circadian rest-activity pattern and cognitive decline, we performed individual linear models including each variable of the rest-activity rhythm (interdaily stability, intradaily variability, and relative amplitude) as predictors and the MMSE at 12 months of follow-up as the outcome. The models were adjusted by age, sex, T-tau/Aβ42 ratio, educational level, and season of the year. The analyses were performed with the global population and stratified by amyloid groups (Aβ42+ and Aβ42− patients). Additional analyses were performed to evaluate the possible effect of sex. The linear models were adjusted by age, T-tau/Aβ42 ratio, educational level, and season of the year, and included the global population stratified by sex. The residuals of the models were evaluated for their suitability in relation to the assumptions of the linear regression.

The *p* value threshold defining statistical significance was set at < 0.05. Data management and statistical analyses were performed using R (version 4.0.1).

## Results

### Baseline characteristics

The cohort included 100 patients diagnosed with mild-moderate AD with a median [p_25_;p_75_] of 23.5 [22.0;25.0] on the MMSE (Table 1). The median age was 76.0 [73.0;80.0] years and 63.0% of the patients were female. Common comorbidities were hypertension (59.0%), depression (28.0%), cardiopathy (21.0%), and diabetes mellitus (20.0%). In addition, 41 (41.8%) patients were ApoEε4+. Considering the most recent NIA-AA research framework based on the pathological process [32], we performed additional analyses including only the Aβ42+ patients. Similar characteristics were observed in this subpopulation (Additional file 1).

**Table 1:** Baseline characteristics of the cohort

	Global
	*n*=100
	*n (%) or median [p*_*25*_*;p*_*75*_*]*
***Sociodemographic data***	
Sex	
Female	63 (63.0%)
Male	37 (37.0%)
Age, years	76.0 [73.0;80.0]
BMI, kg·m^−2^	27.6 [25.1;30.6]
Education, years	7.00 [7.00;7.00]
***Comorbidities***	
Hypertension	59 (59.0%)
Diabetes mellitus	20 (20.0%)
Cardiopathy	21 (21.0%)
Depression	28 (28.3%)
Periodic limb movements	44 (49.4%)
***Cognition***	
MMSE	23.5 [22.0;25.0]
***CSF biomarkers***	
Aβ42, pg/ml	533 [402;669]
T-tau, pg/ml	464 [330;598]
P-tau, pg/ml	72.3 [53;93]
***Genetic risk***	
ApoEε4−	41 (41.8%)

*ApoEε4−* apolipoprotein E carrier, *Aβ42* amyloid-beta protein, *BMI* body mass index, *CSF* cerebrospinal fluid, *MMSE* Mini-Mental State Examination, *n* number, *p* percentile, *P-tau* phosphorylated-tau, *T-tau* total-tau. Missings: Depression, 1; Periodic limb movements, 11; CSF biomarkers, 10; ApoEε4−, 2

### Circadian rest-activity pattern and sleep

We calculated the interdaily stability, intradaily variability, and relative amplitude to evaluate the rest-activity rhythm of our population (Table 2). This analysis demonstrated a median [p_25_;p_75_] of 0.57 [0.49;0.63] for interdaily stability, 0.80 [0.68;0.95] for intradaily variability, and 0.88 [0.84;0.92] for the relative amplitude. Most of the population (56.0%) slept less than 7 h per night, with a median of 83.5% [78.1;88.4] of sleep efficiency. Accordingly, there was a median of 39.4 [32.2;52.2] min of WASO, with 80% of the patients spending more than 30 min awake after sleep onset. The circadian rest-activity pattern and the sleep of both subpopulations (Aβ42+ and Aβ42− patients) presented similar characteristics to the global population (Additional file 2).

**Table 2:** Circadian rest-activity pattern and sleep

	Global
	*n*=100
	*n (%) or median [p*_*25*_*;p*_*75*_*]*
***Rest-activity rhythm***	
Interdaily stability	0.57 [0.49;0.63]
Intradaily variability	0.80 [0.68;0.95]
Relative amplitude	0.88 [0.84;0.92]
M10	116 [83.6;151]
L5	6.44 [4.48;9.93]
***Sleep***	
Time in bed, hours	8.98 [8.08;9.57]
Total sleep time, hours	6.70 [5.54;7.56]
< 7 h	56 (56.0%)
7–9 h	39 (39.0%)
> 9 h	5 (5.0%)
Sleep efficiency, %	83.5 [78.1;88.4]
≥ 85%	44 (44.0%)
84–75%	41 (41.0%)
< 75%	15 (15.0%)
Latency, minutes	22.0 [13.8;33.2]
≤ 30 min	69 (69.0%)
31–60 min	25 (25.0%)
> 60 min	6 (6.0%)
WASO, minutes	39.4 [32.2;52.2]
≤ 30 min	20 (20.0%)
> 30 min	80 (80.0%)

*L5* the mean activity of the five consecutive hours with the lowest activity, *M10* the mean activity of the ten consecutive hours with the highest activity, *n* number, *p* percentile, *WASO* time spent awake after sleep onset

### Baseline characteristics and the circadian rest-activity pattern

**Fig. 1:**
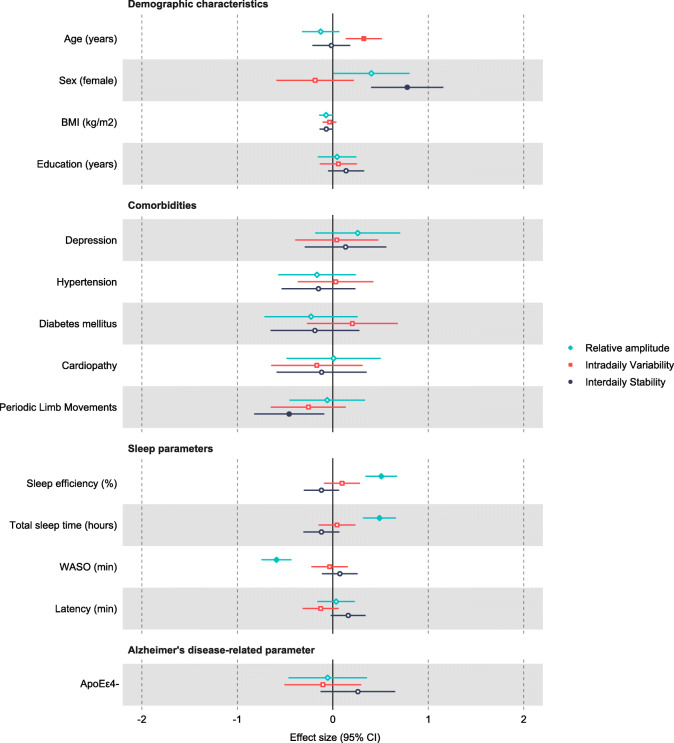
Baseline characteristics according to the circadian rest-activity pattern. The color-filled symbols represent statistically significant associations. ApoEε4-, apolipoprotein E carrier; BMI, body mass index; CI, confidence interval; WASO, time spent awake after sleep onset

Linear regression models were performed to evaluate the baseline characteristics according to the rest-activity rhythm (Fig. 1). We observed an association between increased intradaily variability and higher age with an effect size (SE) of 0.324 (0.096), while increased interdaily stability was associated with female sex with an effect size of 0.780 (0.193). After adjusting for age and sex, there were significant associations between relative amplitude and sleep parameters with an effect size of 0.489 (0.088) for total sleep time, of 0.508 (0.085) for sleep efficiency, and of − 0.590 (0.080) for WASO.

### CSF biomarkers and the circadian rest-activity pattern

Linear regression models adjusted for confounding factors (age, sex, and season of the year) were performed to assess the levels of the CSF biomarkers according to the rest-activity rhythm (Table 3 and Additional file 3). We observed that increased NF-L levels were associated with higher interdaily stability with an effect size [95% CI] of 0.322 [0.076 to 0.569], 0.309 [− 0.003 to 0.620], and 0.603 [− 0.118 to 1.088] in the analyses considering the global population, Aβ42+ patients, and Aβ42- patients, respectively. In addition, increased NF-L levels were associated with higher relative amplitude among the global population and Aβ42+ patients (global: 0.444 [0.212 to 0.676]; Aβ42+ patients: 0.503 [0.209 to 0.797]), but the effect size was reduced among Aβ42− patients (0.186 [− 0.330 to 0.701]). Higher levels of T-tau were associated with increased relative amplitude in the three populations (global: 0.343 [0.139 to 0.547]; Aβ42+ patients: 0.304 [0.046 to 0.561]; Aβ42− patients: 0.507 [0.124 to 0.890]). Additional analyses revealed similar results between males and females (Additional file 4).

**Table 3:** CSF biomarkers according to the circadian rest-activity pattern

Biomarkers	Intradaily variability		Interdaily stability		Relative amplitude	
	*Effect size (95% CI)*	*p value*	*Effect size (95% CI)*	*p value*	*Effect size (95% CI)*	*p value*
***Aβ42***						
Global	0.067 (− 0.144 to 0.277)	0.529	− 0.177 (− 0.391 to 0.038)	0.106	− 0.192 (− 0.405 to 0.020)	0.076
Aβ42+	− 0.150 (− 0.337 to 0.037)	0.114	− 0.042 (− 0.231 to 0.147)	0.660	− 0.010 (− 0.195 to 0.175)	0.910
***T-tau***						
Global	− 0.159 (− 0.367 to 0.050)	0.133	0.109 (− 0.109 to 0.326)	0.322	0.343 (0.139 to 0.547)	0.001
Aβ42+	− 0.034 (− 0.315 to 0.247)	0.809	0.172 (− 0.102 to 0.445)	0.213	0.304 (0.046 to 0.561)	0.022
***P-tau***						
Global	− 0.089 (− 0.300 to 0.122)	0.404	0.068 (− 0.151 to 0.286)	0.540	0.205 (− 0.008 to 0.418)	0.059
Aβ42+	0.071 (− 0.228 to 0.370)	0.636	0.095 (− 0.200 to 0.390)	0.521	0.114 (− 0.174 to 0.401)	0.431
***NF***− ***L***						
Global	− 0.056 (− 0.298 to 0.185)	0.642	0.322 (0.076 to 0.569)	0.011	0.444 (0.212 to 0.676)	0.001
Aβ42+	− 0.070 (− 0.389 to 0.250)	0.662	0.309 (− 0.003 to 0.620)	0.052	0.503 (0.209 to 0.797)	0.001

Linear regression models adjusted by age, sex, and season of the year were performed to assess the biomarkers levels according to the circadian rest-activity pattern

*Aβ42* amyloid-beta protein, *CI* confidence interval, *NF-L* neurofilament light, *P-tau* phosphorylated-tau, *T-tau* total-tau

### Cognitive decline and the circadian rest-activity pattern

**Fig. 2:**
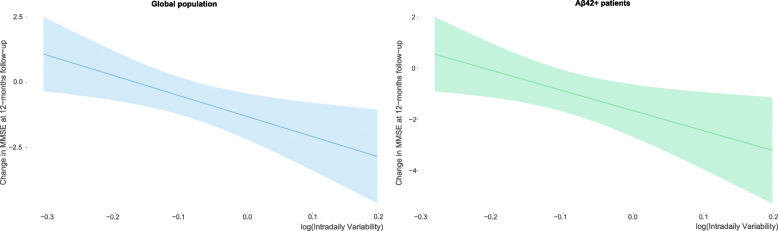
Cognitive decline according to the fragmentation of the rest-activity rhythm considering the global population or Aβ42+ patients only. Intradaily variability was log-transformed to normalize its distribution. MMSE, Mini-mental state examination

We performed linear regression models to assess the cognitive decline according to the rest-activity rhythm (Table 4). A higher intradaily variability predicted an increased cognitive decline at one year of follow-up (effect size [95% CI] of -0.715 [-1.272 to -0.157]) after adjusting for age, sex, T-tau/Aβ42 ratio, educational level, and season of the year (Fig. 2). The analysis including only Aβ42+ patients presented a similar effect size (− 0.717 [− 1.391 to − 0.042]), which was not maintained when considering only Aβ42− patients (− 0.579 [− 2.054 to 0.895]) (Additional files 5 and 6). Additional analyses suggested an effect of the sex within this context (Additional file 7). No associations were observed in relation to the interdaily stability and relative amplitude.

**Table 4:** Cognitive decline according to the circadian rest-activity pattern

	Model 1		Model 2	
	*Effect size (95% CI)*	*p value*	*Effect size (95% CI)*	*p value*
***Intradaily variability***				
Global	− 0.665 (− 1.190 to − 0.140)	0.014	− 0.715 (− 1.272 to − 0.157)	0.013
Aβ42+	− 0.522 (− 1.147 to 0.103)	0.100	− 0.717 (− 1.391 to − 0.042)	0.038
***Interdaily stability***				
Global	− 0.266 (− 0.818 to 0.286)	0.341	− 0.205 (− 0.817 to 0.407)	0.507
Aβ42+	− 0.108 (− 0.778 to 0.562)	0.747	0.009 (− 0.690 to 0.708)	0.979
***Relative amplitude***				
Global	− 0.495 (− 1.025 to 0.036)	0.067	− 0.587 (− 1.192 to 0.019)	0.057
Aβ42+	− 0.658 (− 1.278 to − 0.038)	0.038	− 0.520 (− 1.202 to 0.163)	0.132

Linear regression models were performed to assess the cognitive decline according to the circadian rest-activity pattern. Model 1, unadjusted analysis; model 2, adjusted for age, sex, T-tau/Aβ42 ratio, educational level, and season of the year

*Aβ42* amyloid-beta protein, *CI* confidence interval, *T-tau* total-tau

## Discussion

In the current study, we performed a comprehensive evaluation of the circadian rest-activity pattern of patients with mild-moderate AD. The analysis demonstrated an association between older age and increased fragmentation of the rhythm. Female sex was associated with higher interdaily stability, suggesting increased synchronization between the endogenous rhythm and the zeitgebers in this subgroup of patients. Furthermore, fragmentation of the rhythm at baseline was associated with greater cognitive decline after one year of follow-up, suggesting a predictive role of the rest-activity pattern for the cognition of mild-moderate AD patients. Finally, we evaluated whether the rest-activity pattern was associated with different markers related to the disease’s physiopathology, including Aβ42, P-tau, T-tau, and NF-L. This analysis revealed associations especially related to the relative amplitude.

The increased fragmentation of the rest-activity rhythm in older adults was previously demonstrated in healthy individuals [10, 33, 34] and in subjects with diabetic complications [35], cancer [36], heart failure [37], and AD pathology without clinical symptoms [10]. In this study, we demonstrated that a similar relationship is also present in patients with mild-moderate AD. In contrast, we did not observe an association between interdaily stability and age. Several studies show divergent findings in this regard, reporting higher [33, 34], lower [8], or unchanged [10] synchronization between the endogenous rhythm and the zeitgebers in older adults, which may vary according to different factors such as institutionalization, retirement, and the presence of chronic diseases. There was an association between higher interdaily stability and female sex, corroborating previous findings [33, 38]. In fact, male individuals appear to be more vulnerable to circadian desynchronization induced by inappropriate light exposure [38], which is frequent among AD patients [39].

A previous study demonstrated increased fragmentation of the rest-activity rhythm in cognitively normal individuals with preclinical amyloid plaque pathology, indicating that circadian dysfunction could be a marker for the preclinical stage and/or contribute to the pathogenesis of the disease [10]. Accordingly, healthy older adults with lower amplitude and higher fragmentation of the rhythm demonstrated an increased risk of developing Alzheimer’s dementia [8]. Additionally, individuals with mild cognitive impairment and high intradaily variability presented an increased risk of conversion to AD compared with those with lower fragmentation [8]. Here, we demonstrated that increased intradaily variability at baseline was associated with greater cognitive decline after one year of follow-up, especially in males. This suggests a predictive role of the rest-activity pattern for cognition once individuals are already diagnosed with AD. Although the relationship between circadian function and disease progression appears to be bidirectional [8, 12, 40], some studies suggest mechanisms by which circadian alterations could foster cognitive decline. High fragmentation of the rest-activity rhythm is a consequence of increased periods of rest during the active phase and/or increased periods of activity during the rest phase, which ultimately affects sleep quality. Sleep is an important process for memory consolidation [41] and sleep deprivation or pharmacologically induced wakefulness increase the levels of Aβ42 [42, 43]. Thus, the increased cognitive decline in patients with higher fragmentation of the rest-activity rhythm could be, at least in part, a consequence of inappropriate sleep quality. Furthermore, a significant number of molecules associated with memory consolidation processes and with the pathophysiology of AD are under circadian control. Considering this, it is plausible to expect the fragmentation of the rest-activity rhythm to influence the cognitive decline [44, 45]. Further studies will be necessary to investigate whether a relationship of causality between the circadian rest-activity pattern and cognitive decline is present and the possible mechanisms underlying this. Also, further evaluations will be necessary to confirm the possible differential effect of the sex within this context.

Our results showed an unexpected association between the amplitude of the rhythm and biomarkers such as T-tau and NF-L [46–48]. The relative amplitude represents the robustness of the rest-activity rhythm; hence, one would expect decreased levels of these biomarkers in patients with higher amplitude. In this regard, it is important to consider that both events could be simultaneously occurring as consequences of different processes without any causality between them. Additionally, these findings should be interpreted considering the particularities of this population. A higher level of activity in AD patients during the day (contributing to higher amplitude) may indicate increased agitation and mental confusion, which is expected in more advanced cases with supposedly higher levels of T-tau and NF-L [48, 49]. Also, the observed association between NF-L and interdaily stability should be pondered. Institutionalized individuals are assigned to external schedules, which may wrongly suggest good stability of the rhythm [8, 39]. Even though none of the patients were institutionalized neither at baseline nor after one year of follow-up, they may be under the influence of relatives’ schedules at an early stage of the disease. Further studies with a prospective design and with molecular markers of circadian function will be necessary to improve our understanding of this matter.

## Limitations

The first limitation of this study is that the patients were enrolled from a cognitive unit, and not from a population-based community. Second, the observed associations between circadian rest-activity pattern and cognitive decline were exclusively based on the MMSE, preventing a detailed identification of the relevant cognitive domains in this regard. Third, given the low prevalence of males in our sample, the findings suggesting a differential effect of the sex on the investigated associations should be considered with caution and confirmed in future studies. Fourth, considering the observational design of this study, it is not possible to establish directionality between the associations of interest. Fifth, the measurements performed with actigraphy are influenced by physical activity. Other circadian markers in addition to the rest-activity rhythm should be considered to confirm our findings in future studies. Finally, it is important to address that only patients with mild-moderate AD were included in the analyses and therefore the results herein presented should be limited to this specific population. The observed associations might change according to the stage of the disease or contexts other than AD.

## Conclusions

In summary, we observed that older age and male sex were associated with worse outcomes in terms of rest-activity patterns in a population of mild-moderate AD patients. Accordingly, older age was associated with increased fragmentation of the rhythm, while male sex was related to lower synchronization between the endogenous rhythm and the zeitgebers. In addition, increased fragmentation of the rhythm at baseline was associated with greater cognitive decline after one year of follow-up. This finding sets the rest-activity pattern as a potential predictor of cognitive decline in mild-moderate AD patients. Further studies are warranted to confirm our findings and to improve our understanding of the possible association between the rest-activity pattern and CSF biomarkers once the individuals are diagnosed with the disease.

## Supplementary Information

**Additional file 1 **: **Suppl Table 1**. Baseline characteristics of Aβ42+ and Aβ42- patients.

**Additional file 2 **: **Suppl Table 2**. Circadian rest-activity pattern and sleep of Aβ42+ and Aβ42- patients.

**Additional file 3 **: **Suppl Table 3**. CSF biomarkers according to the circadian rest-activity pattern in Aβ42- patients.

**Additional file 4 **: **Suppl Table 4**. CSF biomarkers according to the circadian rest-activity pattern in males and females.

**Additional file 5 **: **Suppl Table 5**. Cognitive decline according to the circadian rest-activity pattern in Aβ42- patients.

**Additional file 6 **: **Suppl Figure 1**. Cognitive decline according to the fragmentation of the rest-activity rhythm in Aβ42- patients.

**Additional file 7 **: **Suppl Table 6**. Cognitive decline according to the circadian rest-activity pattern in males and females.

## Data Availability

The datasets used and/or analyzed during the current study are available from the corresponding author on reasonable request.

## Abbreviations

AD: Alzheimer’s disease
ApoE: Apolipoprotein E
Aβ42: Amyloid-beta protein
BMI: Body mass index
CI: Confidence interval
CSF: Cerebrospinal fluid
MMSE: Mini-Mental State Examination
NF-L: Neurofilament light
P-tau: Phosphorylated-tau
SE: Standard error of mean
T-tau: Total tau
WASO: Wake after sleep onset
