# Supplementary figures and images for: The circadian rest-activity pattern predicts cognitive decline among mild-moderate Alzheimer’s disease patients
Source: Alzheimers Res Ther. 2021 Sep 25;13:161. doi: 10.1186/s13195-021-00903-7 (PMC8466995; doi:10.1186/s13195-021-00903-7)

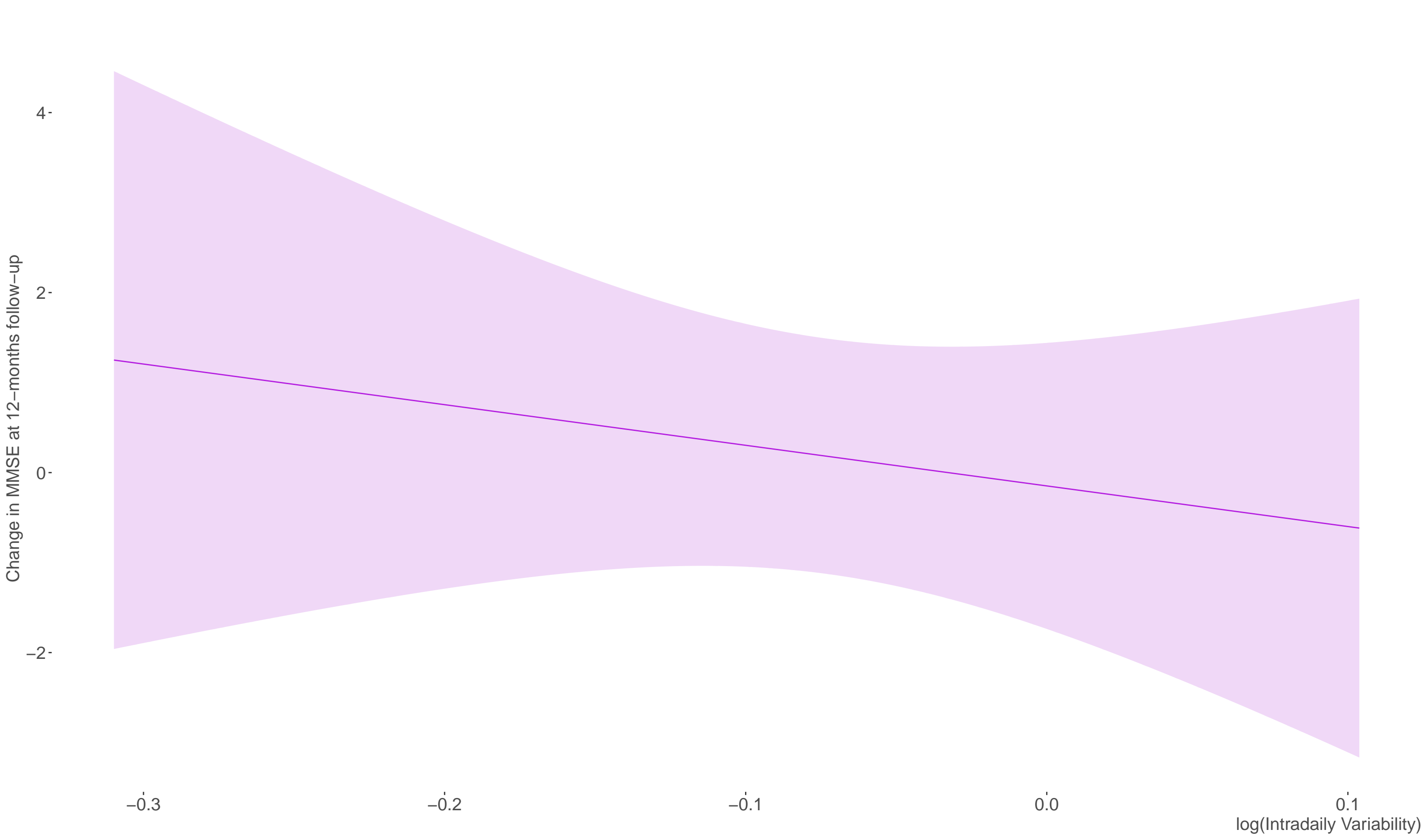

Supplement: Supplementary file 6 — Additional file 6 : Suppl Figure 1. Cognitive decline according to the fragmentation of the rest-activity rhythm in Aβ42- patients. [file 13195_2021_903_MOESM6_ESM.pdf]
